# Seasonal Variability and Pathogenicity of Kiwifruit Bacterial Canker Pathogens in Sichuan Province, China

**DOI:** 10.3390/pathogens14020191

**Published:** 2025-02-14

**Authors:** Chengyong He, Zihong Xu, Lingli Wang, Yarui Li, Jing Li, Haiyan Song, Dong Chen, Guoliang Jiang, Meiyan Tu

**Affiliations:** 1 Institute of Horticulture, Sichuan Academy of Agricultural Sciences, Chengdu 610066, China; hechyong@scsaas.cn (C.H.); garysky@126.com (Z.X.); wanglingli0306@scsaas.cn (L.W.); lyrui1920@126.com (Y.L.); lijing@scsaas.cn (J.L.); lovesong@scsaas.cn (H.S.); chendong@scsaas.cn (D.C.); jiangguoliang@scsaas.cn (G.J.); 2 Key Laboratory of Horticultural Crop Biology and Germplasm Creation in Southwest China, Ministry of Agriculture and Rural Affairs, Chengdu 610066, China

**Keywords:** kiwifruit, Psa, summer canker disease, Pca

## Abstract

Kiwifruit canker disease, caused by different bacterial pathogens, was observed in Sichuan Province, China. Specifically, in the winter and spring seasons, *Pseudomonas syringae* pv. *actinidiae* (Psa) was identified as the primary pathogen, causing reddish-brown exudates, branch dieback, and phloem decay. In contrast, during the summer months, *Pectobacterium carotovorum* subsp. *actinidiae* (Pca) emerged as the primary causal agent of kiwifruit canker, exhibiting similar symptoms to those caused by Psa, such as exudates from leaf scars and lenticels, xylem necrosis, and branch desiccation. From 55 symptomatic samples, 34 bacterial isolates were obtained, with 28 identified as Psa and 6 as Pca. Pathogenicity tests revealed significant variation in virulence, with Psa isolate G5 and Pca isolate M5 showing the highest pathogenicity on leaves and branches, respectively. Both pathogens caused characteristic necrosis and lesion expansion, fulfilling Koch’s postulates. Phylogenetic analyses confirmed the distinct evolutionary relationships of Psa and Pca isolates. These findings highlight the seasonal variability of kiwifruit canker pathogens and emphasize the need for targeted disease management strategies.

## 1. Introduction

Kiwifruit, renowned for its unique taste and nutritional value, is a globally popular fruit, especially among consumers in China and New Zealand. China is not only the native land of kiwifruit but also ranks first in the world in terms of cultivation area and production volume, and it is the largest consumer of kiwifruit as well. However, kiwifruit cultivation encounters numerous challenges, including various diseases that can significantly impact the yield and quality of the fruit. Among these, bacterial canker stands out as a significant threat to kiwifruit production.

Kiwifruit bacterial canker, caused by *Pseudomonas syringae* pv. *actinidiae* (Psa), was first identified on kiwifruit in Shizuoka Prefecture, Japan, in 1984 [1]. Since then, it has spread to various major kiwifruit-producing countries worldwide, including Italy [2], Spain [3], France [4], Portugal [5], New Zealand [6,7], and China [8]. Currently, five bio-types of Psa have been identified globally [9], with the highly pathogenic Psa biovar 3 being the predominant strain in China [10]. Kiwifruit bacterial canker is a devastating disease that has the potential to impact multiple parts of the plant, including its trunk, branches, leaves, buds, and fruits [11]. When the trunk and branches are infected, early symptoms include the appearance of water-soaked patches accompanied by a sticky, white substance. This fluid oxidizes and turns into a reddish-brown color when exposed to air for an extended period. As the disease advances, the bark starts to peel away from the xylem, leading to swelling and the development of prominent cankers in the infected area. Additionally, longitudinal cracks appear within the bark, disrupting the flow of nutrients and water. In severe cases, these lesions can completely encircle the branches, ultimately causing the tree to die [12].

Leaves infected with kiwifruit canker initially show water-soaked lesions, which gradually enlarge to form irregular brown lesions bordered by yellow halos. As the disease progresses, the leaves develop a burnt appearance, curl up, and eventually fall off the plant. Buds infected with this disease often fail to bloom properly and subsequently turn brown and wither. Even if a small percentage of infected buds manage to flower, they may not produce fruit or may yield misshapen fruits.

The kiwifruit summer canker disease, caused by *Pectobacterium carotovorum* subsp. *actinidiae* (Pca), has been documented in only a handful of locations to date, namely, South Korea [13] and specific regions in China such as Sichuan [14], Jiangxi [15], Zhejiang [16], and Guangxi [17]. The typical symptoms induced by Pca include the appearance of red discoloration on infected branches or trunks, along with the seepage of mucus. Subsequently, the leaves wither, the shoots dry up and die, and in extreme cases, the entire plant may ultimately succumb to the disease. Based on current research findings, the extent of its devastation remains relatively modest. When the disease was first detected in Sichuan, symptoms were endemic in various townships with sporadic outbreaks, but 3 to 5% of infected plants were observed in three orchards in the town of Daxing [14]. In Fengxin City, Jiangxi Province, the incidence rate stood at approximately 10% [15]. In contrast, in a 15-hectare orchard situated in Lishui City, Zhejiang Province, only about 3% of the strains were found to be infected [15]. This particular disease presents symptoms that are strikingly similar to those of the kiwifruit canker disease induced by Psa. The pathogen thrives best at temperatures hovering around 30 °C, and it particularly favors environments characterized by high temperatures and humidity, conditions under which it multiplies rapidly [13].

To accurately identify the pathogenic bacteria causing kiwifruit canker disease in Sichuan Province during different seasons, this study collected samples in 2023–2024, followed by systematic isolation and identification processes. The results indicate that the pathogen of kiwifruit canker in winter and spring is *Pseudomonas syringae* pv. *actinidiae*, whereas in summer, causative agent transitions to *Pectobacterium carotovorum* subsp. *actinidiae*. These findings have significantly enhanced our comprehension of kiwifruit bacterial canker while simultaneously establishing a foundation for the precise prevention and control of the disease throughout various seasons.

## 2. Materials and Methods

### 2.1. Sample Collection

During the period from December 2023 to March 2024, a total of 9 trunks, 11 branches, and 4 leaves exhibiting typical symptoms of bacterial canker were collected from 6 orchards in Guangyuan City and Dujiangyan City, Sichuan. Subsequently, in August 2024, an additional 6 samples of branches suspected of having kiwifruit canker disease were further collected from an orchard located in Leshan City, Sichuan. To ensure the freshness and integrity of the samples, all collected samples were properly stored in foam boxes with ice packs and promptly transported to the laboratory, where they were kept in refrigerators at 4 °C for subsequent pathogen isolation and identification procedures.

### 2.2. Isolation of Pathogenic Bacteria

The collected samples were initially rinsed with tap water to remove impurities and then carefully wiped clean. On a benchtop, sterile blades were used to excise tissues from the junction between diseased and healthy tissue. These tissue fragments were sequentially immersed in 75% ethanol for 30 s and then disinfected in 1% sodium hypochlorite for 5 min, with each step followed by three rinses with sterile water. Subsequently, the tissue fragments were placed in centrifuge tubes filled with sterile water, and a sterile glass rod was employed to thoroughly macerate the samples. After allowing the mixture to stand for 10 min, the suspension was serially diluted in three concentration gradients, each diluted 100 times. Using a pipette, 100 μL aliquots from each dilution gradient were plated onto LB solid medium. The plates were then inverted and incubated in a 28 °C incubator. After 48 h, individual colonies were picked and streaked onto LB solid medium to obtain purified cultures.

### 2.3. DNA Extraction

We dispensed 50 μL of Lysis Buffer for Microorganisms to Direct PCR (TaKaRa, Dalian, China) into a sterile microtube, selected a single colony using a sterile toothpick and gently swirled it in the microtube a couple of times before withdrawing it. Subsequently, we subjected the mixture to thermal denaturation at 80 °C for 15 min. Following the denaturation process, we conducted a low-speed centrifugation until the DNA was dissolved in the lysis buffer.

### 2.4. PCR, Cloning, and Sequencing

Bacterial DNA served as the template for PCR amplification, utilizing high-fidelity Pfu DNA polymerase (Tiangen, Beijing, China). The PCR reaction mixture comprised 2.5 mM dNTPs, 2 × Reaction buffer, 2.0 mM MgCl_2_, and specific primers 16S-F/R, Psa-F/R, and Pca-F/R (Table S1). The PCR protocol involved an initial denaturation step at 98 °C for 1 min, followed by 35 amplification cycles, each consisting of denaturation at 98 °C for 10 s, annealing at 55 °C for 15 s, and extension at 72 °C for 20 s. A final extension step was conducted at 72 °C for 5 min, utilizing a Mastercycler thermal cycler (Eppendorf, Hamburg, Germany).The PCR products underwent electrophoresis on a 1.5% agarose gel and were subsequently purified using the AxyPrep™ DNA Gel Extraction Kits (Axygen, Silicon Valley, CA, USA). The purified products were then ligated into the pTOPO vector (Aidelab, Beijing, China), and the resulting recombinant plasmids were transformed into *Escherichia coli* DH5α cells, and positive clones were randomly picked for sequencing.

### 2.5. Preparation of Pathogenic Bacterial Suspension

The verified and ultra-low temperature-stored Psa and Pca bacterial suspension were retrieved and streaked onto LB plates on a benchtop. After activation and incubation at 25 °C for a period of 48 h, a single colony was randomly picked and subjected to colony PCR verification utilizing either Psa or Pca specific primers as described in Section 2.4. Positive clones were inoculated into liquid LB and then concentrated by centrifuging at 3000× *g* for 5 min, washed thoroughly three times with sterile water, and adjusted to an OD_600_ = 0.1.

### 2.6. Pathogenicity Analysis

The prepared bacterial suspension was used to inoculate kiwifruit plants as described previously [16]. For inoculating branches, the one-year-old branches of the ‘Hongyang’, stored in a freezer, were first disinfected with 75% alcohol for 30 s and then placed in a 0.5% sodium hypochlorite solution for an additional 10 min. This was followed by thorough rinsing with sterilized distilled water for approximately 15 min each time, with this rinsing process being repeated 3 to 4 times to guarantee the complete removal of any residual disinfectant. After naturally air-drying, the branches were cut into short sections of approximately 15 cm in length, and the ends of each section were sealed with paraffin. Next, a 1 mm wide wound was made on the surface of each branch, with the depth extending to the phloem. Then, 10 μL of bacterial suspension, with an OD600 nm value of 0.1, was carefully dropped into the wound. For comparative purposes, a control group was established where an equal amount of sterile water was dropped onto the wounds of the branches.

For inoculating leaves, we chose fully expanded, newborn ‘Hongyang’ leaves that exhibited uniform growth and size. We disinfected these leaves using a 0.5% sodium hypochlorite solution for a duration of 5 min. Following this, we rinsed them meticulously with sterile water and subsequently used filter paper to blot away any lingering moisture from the leaf surface. Afterward, we evenly sprayed the leaves with a bacterial suspension that contained a concentration of 10^6^ cfu/mL.

Once the inoculation process was completed, the branches and leaves were placed carefully in trays containing an appropriate amount of sterile water to maintain the necessary humidity. The trays were then covered with film to further retain moisture and create an optimal environment for growth. Finally, the entire tray was placed in an artificial climate incubator, with the cultivation conditions set to a photoperiod of 16 h of light and 8 h of darkness, at a constant temperature of 16 °C.

### 2.7. Bioinformatics Analyses

The ClustalW method was applied to multiple sequence alignments, and a phylogenetic tree was constructed using the Neighbor-Joining method in MEGA 11 [17], with a bootstrap of 1000 replicates.

## 3. Results

### 3.1. Field Disease Investigation

**Figure 1:**
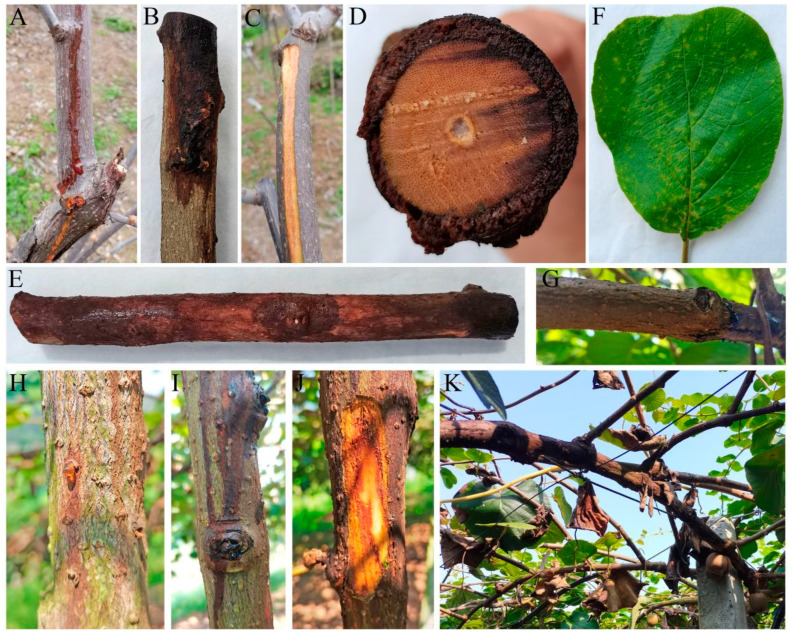
Disease symptoms in the field: (**A**–**C**) kiwifruit samples were collected in December 2023 from Cangxi County, Guangyuan City; (**D**–**F**) kiwifruit samples were collected from Dujiangyan City in March 2024; (**G**–**K**) kiwifruit samples were collected in August 2024 from Mabian County, Leshan City.

In December 2023, kiwifruit branches in an orchard in Cangxi County, Guangyuan City, Sichuan Province, were observed to exhibit symptoms of canker disease. The primary symptoms included reddish-brown bacterial exudates oozing from branch wounds and flowing downward along the branches (Figure 1A). Some of the exudates dried to form dark-brown to black residues that adhered to the stem surface (Figure 1B). Upon peeling off the bark from the affected areas, the underlying wood was found to have a reddish-brown discoloration (Figure 1C). Among the 150 trees surveyed, 10 trees were found to be infected, resulting in a disease incidence rate of approximately 6.7%. We observed kiwifruit branch dieback caused by canker disease in Dujiangyan City in March 2024. The phloem tissue became soft and decayed, with the infection extending into the xylem (Figure 1D), and the affected branches were encased in bacterial exudates (Figure 1E). Irregular brown lesions appeared on the leaves, accompanied by distinct yellow halos surrounding the lesions (Figure 1F). Among the 330 trees surveyed, 18 trees were found to be infected, resulting in a disease incidence rate of approximately 5.5%.

In August 2024, kiwifruit plants in Mabian County, Leshan City, Sichuan Province, exhibited symptoms resembling canker disease. Reddish-brown, translucent bacterial exudates were observed oozing from leaf scars (Figure 1G) and lenticels (Figure 1H). Upon drying, these exudates turned brown or black (Figure 1I). Necrosis and browning were evident in the xylem (Figure 1J), and some branches exhibited desiccation and dieback (Figure 1K). Out of a total of approximately 232 trees in the entire orchard, only 6 trees were found to be infected, resulting in a disease incidence rate of approximately 2.59%.

### 3.2. Bacterial Isolation and Phenotypic Characterization

In total, 55 samples of typical symptomatic kiwifruit trunks, branches, and leaves were collected from three regions in Sichuan Province, China (Figure 1). Using the method of serial streak plate isolation and purification for monoclonal cultures, we successfully obtained 34 isolates from the aforementioned samples. Among them, 18 were isolated from Dujiangyan, 10 from Guangyuan, and the remaining 6 from Leshan. The colony morphology exhibited a milky white color, round shape, smooth texture, and translucent quality, along with a slightly convex appearance.

Using the DNA of 34 strains as templates, PCR amplification was performed with universal primers (Table S1) for 16S rRNA, resulting in amplified fragments of approximately 1500 bp for all samples. The sequence analysis of PCR products indicates that the isolates from Dujiangyan and Guangyuan are both *Pseudomonas syringae* pv. *actinidiae*, whereas the isolates from Leshan are *Pectobacterium carotovorum* subsp. *actinidiae* (Table 1).

### 3.3. Phylogenetic Relationship Between Psa and Pca in This Study and Other Isolates

**Figure 2:**
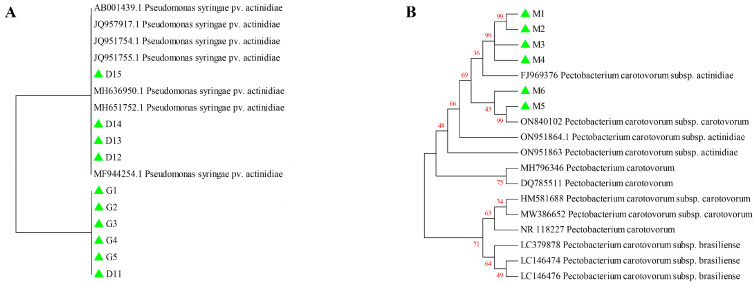
Phylogenetic relationship of 16S rRNA of Psa (**A**) and Pca (**B**) and other isolates from different countries and hosts, based on the nucleotide sequence. The phylogenetic tree was constructed using the Neighbor-Joining method in MEGA 11, with a bootstrap of 1000 replicates. The green triangle indicates the Psa isolated in this study. The red numbers indicate the bootstrap values.

Upon sequence alignment, it was identified that the 16S rRNA sequences of 28 Psa isolates were identical, leading to the selection of ten isolates as the sequence for further phylogenetic analysis. Multiple alignments of 16S rRNA sequences of different *Pseudomonas syringae* isolates were performed using ClustalW, and then, the phylogenetic trees were constructed using the maximum likelihood method with a bootstrap of 1000 replicates in MEGA 11. A phylogenetic analysis revealed that all isolates could be categorized into two primary clades. Specifically, D12, D13, D14, and D15, which were isolated in this study, exhibited a close relationship to other *Pseudomonas syringae* pv. *actinidiae* isolates. Conversely, the isolates G1, G2, G3, G4, G5, and D11 were grouped together (Figure 2A).

To clarify the evolutionary relationships of Pca isolates in this study, multiple alignments of 16S rRNA sequences of different *Pectobacterium carotovorum* isolates were performed. A phylogenetic analysis indicated that M1 to M6 were closely related to *Pectobacterium carotovorum* subsp. *actinidiae* (FJ969376 and ON840102), and were grouped into the same clade, but more distantly related to other *Pectobacterium carotovorum* isolates (Figure 2B).

### 3.4. PCR Amplification of Psa and Pca

To obtain the distinct sequence of Psa and Pca, specific primers Psa-F/R and Pca-F/R (referenced in Table S1) were designed, PCR was performed using DNA from a subset of isolates, and the amplified fragments were then sequenced. PCR amplification of all Psa samples consistently yielded a 280 bp fragment, with sequence alignment confirming complete identity across all sequences. NCBI BLAST analysis indicated that these sequences shared 99.15% to 100% similarity with reference Psa isolates. Similarly, PCR amplification of all Pca samples produced a 343 bp fragment, and sequence alignment revealed identical sequences among the six samples. NCBI BLAST results showed 99.11% to 100% similarity with reference Pca isolates.

### 3.5. Pathogenicity Tests

**Figure 3:**
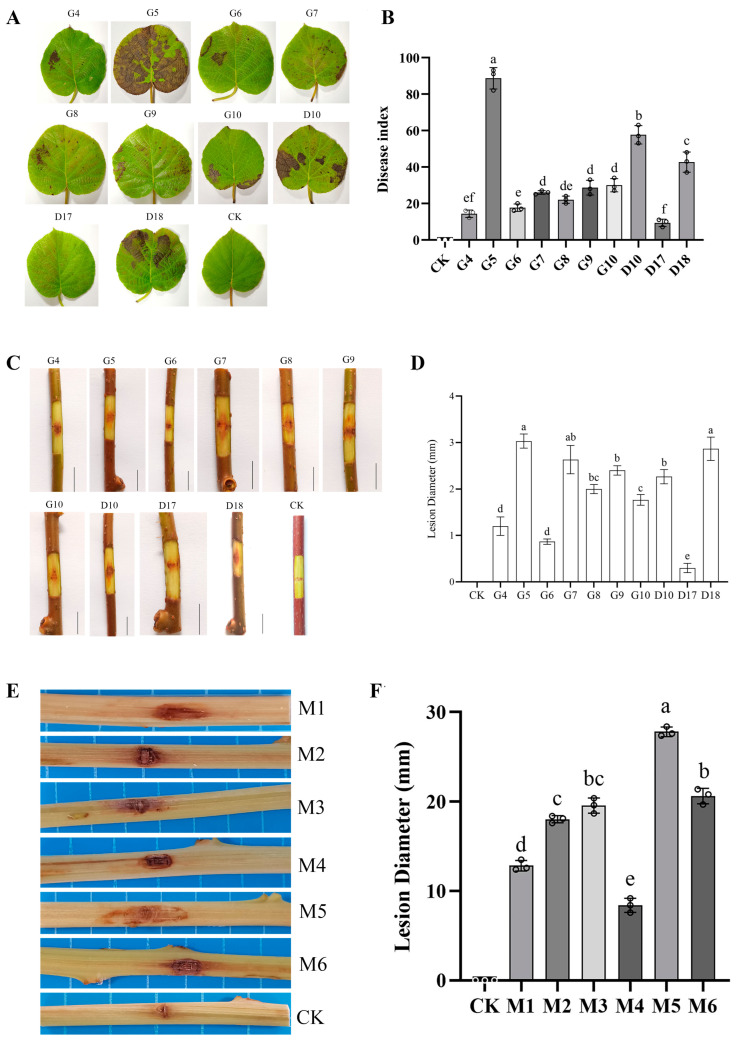
*Pseudomonas syringae* pv. *actinidiae* (Psa) pathogenicity tests: (**A**) the disease symptoms on kiwifruit leaves after inoculating with different isolates of Psa suspensions and water for 6 days under in vitro conditions; (**B**) disease index represents the statistical area of disease lesions; (**C**) the disease symptoms on kiwifruit branch after inoculating with 10 isolates of Psa suspensions and water for 17 days under in vitro conditions; (**D**) lesion diameter was measured to indicate the level of Psa pathogenicity; (**E**) the disease symptoms on kiwifruit branch after inoculating with 10 isolates of Psa suspensions and water for 17 days under in vitro conditions; (**F**) lesion diameter was measured to indicate the level of Pca pathogenicity. CK represents the control group treated with sterile water. Different lowercase letters indicate significant differences in lesion diameter at *p* < 0.05. The experiments were performed independently three times and representative results are shown.

To assess the pathogenicity of the diverse Psa isolates obtained in this study, we randomly selected 10 isolates and inoculated them onto ‘Hongyang’ kiwifruit leaves. The results revealed varying degrees of virulence of these 10 isolates (Figure 3A), D17 exhibited mild symptoms with a disease index of 9.3 on the sixth day after inoculation, indicating that this isolate was relatively less virulent to kiwifruit leaves. In contrast, the other isolates caused significant symptoms as early as the fourth day after inoculation. Notably, G5 demonstrated the highest level of pathogenicity, causing widespread necrosis on the leaves with a disease index of 88.7. Following G5, the isolates exhibited the following pathogenicity levels and corresponding disease indices: D10 (57.6), D18 (42.8), G10 (30.0), G9 (28.7), G7 (26.0), G8 (22.0), G6 (17.6), and G4 (14.3) (Figure 3B).

The pathogenicity results were also evidenced on kiwifruit dormant branches using the wound inoculation method. On the 17th day after inoculation, all Psa isolates exhibited obvious inward depression and necrosis in the phloem at the inoculation site, accompanied by the formation of bacterial exudates within the necrotic area. Meanwhile, the xylem generally showed browning, and the lesion area started from the wound surface and gradually spread towards both ends of the inoculation point (Figure 3C). The relative virulence levels of the Psa isolates were evaluated by measuring the expansion diameter of the lesion. The results were similar to those observed in leaf inoculation, with G5 exhibiting the largest lesion expansion diameter of 3.03 cm, while D17 showed the smallest lesion expansion diameter of only 0.3 cm (Figure 3D).

Next, we evaluated the pathogenicity of six Pca isolates by inoculating them onto Hongyang branches. Similar to the symptoms caused by Psa, all six Pca isolates led to necrosis of the phloem and browning of the xylem at the inoculation sites (Figure 3E). In terms of the lesion diameter, M5 exhibited the largest at 28.5 mm, while M4 had the smallest at merely 8.4 mm (Figure 3F), indicating that M5 had the strongest pathogenicity, while M4 displayed the weakest relative to the other isolates.

Pathogenicity tests were conducted three times under identical conditions, and the pathogen was successfully reisolated and identified from symptomatic areas using the aforementioned methods. Control samples failed to detect the presence of the pathogenic bacteria, thereby fulfilling Koch’s postulates.

## 4. Discussion

Kiwifruit canker disease, as an extremely destructive pathogen, poses a severe challenge to the health and sustainable development of the kiwifruit industry. Sichuan, being the second largest kiwifruit production area in China and simultaneously the world’s largest producer of red-fleshed kiwifruit, underscores the significance of its kiwifruit industry. However, red-fleshed kiwifruit varieties are particularly susceptible to the attack of *Pseudomonas syringae* pv. *actinidiae* (Psa), pushing Sichuan’s kiwifruit industry to the brink of collapse at one point. In recent years, although the promotion of rain-shelter cultivation techniques has, to some extent, mitigated the severity of canker disease, the high cost of constructing rain-shelter facilities has become an insurmountable barrier for many small orchards. Consequently, the incidence of canker disease remains high in those orchards that have not adopted such facilities.

It is worth noting that, unlike Psa’s high pathogenicity on red-fleshed kiwifruit, while *Pectobacterium carotovorum* subsp. *actinidiae* (Pca) also occurs on red-fleshed varieties [18], it has a stronger preference for infecting yellow-fleshed kiwifruit varieties, such as *Actinidia chinensis* cv. Hort16A, as previously reported [13,14,15]. Similarly, this study also detected the presence of Pca on yellow-fleshed kiwifruit. Although both bacteria, Psa and Pca, can cause kiwifruit canker disease and exhibit similar symptoms, there are notable differences in their seasonal occurrence. Our study has revealed that temperature is a pivotal factor influencing the growth and transmission of pathogens. Specifically, we observed that lower temperatures tend to favor the survival and dissemination of Psa, whereas higher temperatures are more conducive to the proliferation of Pca. Indeed, Psa migrates faster at 4 °C than at 16 °C or 25 °C in twigs. However, the optimal temperature for the colonization and movement of Psa within leaf veins is 16 °C [19]. In contrast, Pca primarily emerges during the hotter periods of summer, with temperatures of 28–30 °C being more conducive to disease development [20]. In addition to temperature, humidity also emerges as a significant determinant of pathogen behavior. High humidity environments have the potential to accelerate pathogen transmission and enhance infection rates. Moreover, soil conditions, including soil moisture, pH, and nutrient content, play crucial roles in influencing pathogen survival and plant health. Variations in these soil parameters can create favorable or unfavorable environments for pathogens, thereby impacting their prevalence and the overall health of the plant community. Future research should focus on incorporating detailed environmental monitoring and data analysis to better understand the multifaceted factors influencing pathogen prevalence.

Molecular identification through 16S rRNA sequencing, supplemented by specific PCR amplification, verified the presence of 28 Psa isolates from Guangyuan and Dujiangyan, and 6 Pca isolates from Leshan. Phylogenetic analysis demonstrated that the Psa isolates in this study grouped together, exhibiting a high level of similarity (ranging from 99.15% to 100%) with other isolates. The clustering of all Psa isolates into a single clade indicates that these isolates do not exhibit geographical specificity. In contrast, the Pca isolates exhibited greater genetic diversity. Specifically, Pca isolates M1 to M6 grouped closely with the reference strain (FJ969396 and ON840102), whereas isolates ON951863 and ON951864 formed a separate clade, suggesting the presence of geographically differentiated Pca populations. This observed genetic variability among the Pca isolates could indicate local adaptation or independent introductions. Consequently, there is a pressing need for further research into the epidemiology and evolutionary dynamics of Pca.

Pathogenicity assays revealed significant variability in virulence among both Psa and Pca isolates. Among the Psa isolates, G5 exhibited the highest virulence, with a leaf disease index of 88.7 and a lesion expansion diameter of 3.03 cm on branches. Conversely, D17 displayed notably reduced virulence, with a leaf disease index of only 9.3 and a branch lesion diameter of 0.3 cm. In the case of Pca, isolate M5 was the most aggressive, causing a lesion diameter of 28.5 mm on inoculated branches, whereas M4 showed the lowest pathogenicity, with a lesion diameter of 8.4 mm. These differences likely reflect the genetic variability among the isolates. Such variability in pathogenicity further emphasizes the importance of identifying and focusing on the most virulent strains in disease management strategies.

In terms of disease control, the use of resistant cultivars represents a promising strategy for mitigating the impact of both Psa and Pca. Breeding programs should focus on incorporating genetic resistance to these pathogens into commercial varieties. Additionally, biocontrol agents, such as antagonistic bacteria or fungi, could be explored as alternative or supplementary control methods [21]. These agents have shown potential in managing other plant diseases and warrant further investigation for their efficacy against kiwifruit canker. Chemical controls may also play a role in disease management, particularly in situations where resistant cultivars and biocontrol agents are not yet available or fully effective. However, the use of chemicals must be balanced with concerns over environmental impact and the development of pathogen resistance. Therefore, an integrated approach combining resistant cultivars, biocontrol agents, and chemical controls, tailored to the specific needs and conditions of different kiwifruit-producing regions, would be ideal.

This study elucidates the complex epidemiological dynamics and genetic diversity of kiwifruit canker pathogens in Sichuan Province. The seasonal variability in pathogen prevalence, combined with differences in virulence among isolates, underscores the importance of tailored management approaches. Future research should focus on exploring the molecular interactions between kiwifruit hosts and pathogens, the environmental factors driving pathogen evolution, and the development of integrated control strategies to safeguard kiwifruit production in affected regions.

## 5. Conclusions

This study investigated the occurrence and characteristics of kiwifruit canker disease in Sichuan Province. In Guangyuan and Dujiangyan during winter and spring, *Pseudomonas syringae* pv. *actinidiae* was the main pathogen. In Leshan during summer, *Pectobacterium carotovorum* subsp. *actinidiae* was associated with symptoms. A total of 34 bacterial isolates were obtained from 55 symptomatic samples, with 28 identified as Psa and 6 as Pca. Phylogenetic analyses showed distinct evolutionary relationships of these isolates. PCR amplification with specific primers for Psa and Pca gave characteristic fragments with high similarity to reference isolates. Pathogenicity tests revealed significant variation in virulence among the isolates. Psa isolate G5 and Pca isolate M5 showed the highest pathogenicity on leaves and branches, respectively. The fulfillment of Koch’s postulates further confirmed the causal relationship of the pathogens to the disease. These findings highlight the importance of understanding the seasonal variability of kiwifruit canker pathogens, and future disease management strategies should be targeted and adapted to the specific pathogens and seasons to control the disease in Sichuan Province.

## Figures and Tables

**Table 1:** Bacterial strains isolated from diseased kiwifruit samples in Sichuan.

Isolate	Tissue	Cultivar	Region	Bacterial
G1	Trunk	Hongyang	Guangyuan	*Pseudomonas syringae* pv. *actinidiae*
G2	Trunk	Hongyang	Guangyuan	*Pseudomonas syringae* pv. *actinidiae*
G3	Trunk	Hongyang	Guangyuan	*Pseudomonas syringae* pv. *actinidiae*
G4	Branch	Hongyang	Guangyuan	*Pseudomonas syringae* pv. *actinidiae*
G5	Branch	Hongyang	Guangyuan	*Pseudomonas syringae* pv. *actinidiae*
G6	Branch	Hongyang	Guangyuan	*Pseudomonas syringae* pv. *actinidiae*
G7	Branch	Hongyang	Guangyuan	*Pseudomonas syringae* pv. *actinidiae*
G8	Branch	Hongyang	Guangyuan	*Pseudomonas syringae* pv. *actinidiae*
G9	Branch	Hongyang	Guangyuan	*Pseudomonas syringae* pv. *actinidiae*
G10	Branch	Hongyang	Guangyuan	*Pseudomonas syringae* pv. *actinidiae*
D1	Trunk	Hongyang	Dujiangyan	*Pseudomonas syringae* pv. *actinidiae*
D2	Trunk	Hongyang	Dujiangyan	*Pseudomonas syringae* pv. *actinidiae*
D3	Trunk	Ruiyu	Dujiangyan	*Pseudomonas syringae* pv. *actinidiae*
D4	Trunk	Ruiyu	Dujiangyan	*Pseudomonas syringae* pv. *actinidiae*
D5	Trunk	Ruiyu	Dujiangyan	*Pseudomonas syringae* pv. *actinidiae*
D6	Trunk	Ruiyu	Dujiangyan	*Pseudomonas syringae* pv. *actinidiae*
D7	Branch	Hongyang	Dujiangyan	*Pseudomonas syringae* pv. *actinidiae*
D8	Branch	Hongyang	Dujiangyan	*Pseudomonas syringae* pv. *actinidiae*
D9	Branch	Ruiyu	Dujiangyan	*Pseudomonas syringae* pv. *actinidiae*
D10	Branch	Ruiyu	Dujiangyan	*Pseudomonas syringae* pv. *actinidiae*
D11	Leaf	Ruiyu	Dujiangyan	*Pseudomonas syringae* pv. *actinidiae*
D12	Leaf	Ruiyu	Dujiangyan	*Pseudomonas syringae* pv. *actinidiae*
D13	Leaf	Hongyang	Dujiangyan	*Pseudomonas syringae* pv. *actinidiae*
D14	Leaf	Hongyang	Dujiangyan	*Pseudomonas syringae* pv. *actinidiae*
D15	Branch	Ruiyu	Dujiangyan	*Pseudomonas syringae* pv. *actinidiae*
D16	Branch	Ruiyu	Dujiangyan	*Pseudomonas syringae* pv. *actinidiae*
D17	Branch	Ruiyu	Dujiangyan	*Pseudomonas syringae* pv. *actinidiae*
D18	Branch	Ruiyu	Dujiangyan	*Pseudomonas syringae* pv. *actinidiae*
M1	Branch	Hybrid strain	Leshan	*Pectobacterium carotovorum* subsp. *actinidiae*
M2	Branch	Hybrid strain	Leshan	*Pectobacterium carotovorum* subsp. *actinidiae*
M3	Branch	Hybrid strain	Leshan	*Pectobacterium carotovorum* subsp. *actinidiae*
M4	Branch	Hybrid strain	Leshan	*Pectobacterium carotovorum* subsp. *actinidiae*
M5	Branch	Hybrid strain	Leshan	*Pectobacterium carotovorum* subsp. *actinidiae*
M6	Branch	Hybrid strain	Leshan	*Pectobacterium carotovorum* subsp. *actinidiae*

## Data Availability

The data presented in this study are available on request from the corresponding author.

## Supplementary Materials

The following supporting information can be downloaded at https://www.mdpi.com/article/10.3390/pathogens14020191/s1, Table S1: Primers used in this study.
